# Mechanic’s Feet (Hiker’s Feet) in Anti-Synthetase Syndrome

**DOI:** 10.31138/mjr.301023.mfh

**Published:** 2023-01-29

**Authors:** Dogga Prasanna Kumar, Digvijay Ekbote, Kriti Kishor, Urmila Dhakad

**Affiliations:** Department of Clinical Immunology and Rheumatology, King George’s Medical University Lucknow, India

**Keywords:** Mechanic's feet, anti-synthetase syndrome, hyperkeratosis of feet

**Patient 1**: A 37-year-old female presented with complaints of polyarthritis and fissuring of skin on both hands and both feet (non-itchy) for 8 months, dry cough with shortness of breath for 4 months and proximal muscle weakness for 1 month. On examination she had hyperkeratosis with fissuring of skin on radial aspect of all fingers, toes, and plantar surface of feet (**Figure 1A,B**). Chest examination revealed bibasilar crepitations. HRCT was s/o Organising Pneumonia (**Figure 1C**). Work up revealed elevated SGOT>SGPT, CPK and LDH, ANA(IIF) −4+cytoplasmic fine speckled, ENA -Ro 52+ and MSA was anti Jo1 + and was labelled as Anti-synthetase Syndrome.

**Figure 1.**
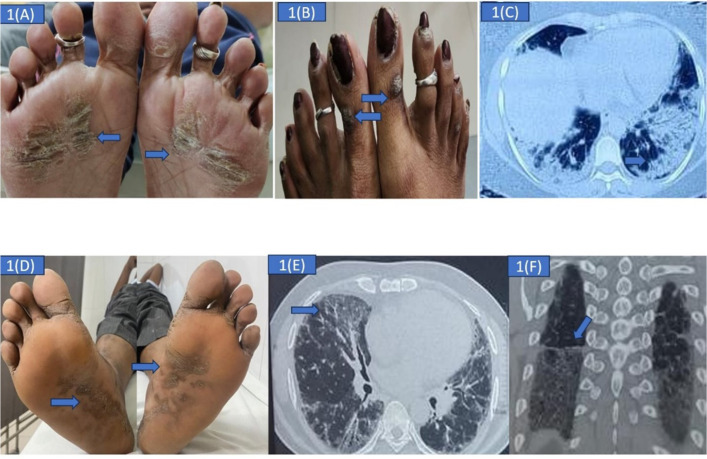
**(A)** Hyperkeratosis, fissuring on the plantar surface of both feet. **(B)** Hyperkeratosis on dorsal aspect of both great toes. **(C)** HRCT Chest s/o Organising Pneumonia pattern of ILD. **(D)** Hyperkeratosis on the plantar surface and medial aspect of both feet. **(E,F)** HRCT Chest s/o NSIP pattern of ILD, anterior upper lobe sign. **(E)** Straight edge sign.

**Patient 2:** A 55-year-old male presented with complaints of Raynaud’s Phenomenon for 1-year, dry cough with progressive shortness of breath for last 6 months, proximal muscle weakness for 2 months and fissuring of both feet (non-itchy). On examination he had fissuring with hyperkeratosis on plantar surface, lateral and medial side of both feet (**Figure 1D**). Chest examination revealed bibasilar crepitations. HRCT was suggestive of NSIP (**Figure 1E,F**).

The work up revealed elevated CPK, LDH, ANA -Negative, ENA -Negative and MSA was anti Jo1 + and was labelled as Anti-synthetase Syndrome.

Mechanic’s hands in Anti-synthetase syndrome is a well-known entity and similar findings (hyperkeratosis and fissuring) on feet now called as hiker’s feet (non-pruritic) is often ignored.^1^ Hiker’s feet is often misdiagnosed as eczema (pruritic) or psoriasis (skin involvement pattern is not limited) or irritant contact dermatitis (h/o handling irritant substances). Recognition of hiker’s feet sign may help in the early suspicion of anti-synthetase syndrome and alert physicians to screen for ILD especially in patients without mechanic’s hands and have only hiker’s feet.^2^ The therapeutic approach is challenging and the choice of therapy with dosing is based on the predominant symptoms at presentation (myositis/ILD/arthritis/skin involvement). Mechanic’s hands and feet respond well to oral steroids plus steroid sparing agents like MMF or rituximab or cyclophosphamide or methotrexate or topical tacrolimus. Arthritis can be erosive, polyarticular, and symmetrical like RA and treated similarly with short course of anti-inflammatory agents (NSAIDs and/or glucocorticoids) until DMARDs like methotrexate or Rituximab take effect.^3^

## References

[1] Stahl NI Klippel JH Decker JL. A cutaneous lesion associated with myositis. Ann Intern Med 1979;91(4):577–9. [PubMed: 484960] 484960 10.7326/0003-4819-91-4-577

[2] Cox JT Gullotti DM Mecoli CA Lahouti AH Albayda J Paik J Johnson C Danoff SK Mammen AL Christopher-Stine L. “Hiker’s feet”: a novel cutaneous finding in the inflammatory myopathies. Clin Rheumatol 2017 Jul; 36:1683–6. 28389987 10.1007/s10067-017-3598-5 PMC6100725

[3] Zanframundo G Marasco E La Carrubba C De Stefano L Volpiano L Tirelli C Update on treatment of anti-synthetase syndrome: A brief review. Curr Treat Options Rheumatol 2020 Mar; 6:18–28.

